# Mitigating the Threat of Invasive Mosquito Species Expansion: A Comprehensive Entomological Surveillance Study on Kastellorizo, a Remote Greek Island

**DOI:** 10.3390/insects15090724

**Published:** 2024-09-20

**Authors:** Marina Bisia, Georgios Balatsos, Stavroula Beleri, Nikolaos Tegos, Evangelia Zavitsanou, Shannon L. LaDeau, Vasilis Sotiroudas, Eleni Patsoula, Antonios Michaelakis

**Affiliations:** 1 Laboratory of Insects and Parasites of Medical Importance, Scientific Directorate of Entomology and Agricultural Zoology, Benaki Phytopathological Institute, 145 61 Kifissia, Greece; m.bisia@bpi.gr (M.B.); g.balatsos@bpi.gr (G.B.); e.zavitsanou@bpi.gr (E.Z.); 2 Laboratory for the Surveillance of Infectious Diseases, Department of Public Health Policy, School of Public Health, University of West Attica, 115 21 Athens, Greece; smpeleri@uniwa.gr (S.B.); ntegos@uniwa.gr (N.T.); epatsoula@uniwa.gr (E.P.); 3 Cary Institute of Ecosystem Studies, Millbrook, NY 12545, USA; ladeaus@caryinstitute.org; 4 AgroSpeCom, 7th klm National Road Thessaloniki-Katerini, Kalochori, 570 09 Thessaloniki, Greece; v.sotiroudas@agrospecom.gr

**Keywords:** Asian tiger mosquito, *Aedes cretinus*, human landing collections, KAP questionnaires

## Abstract

**Simple Summary:**

The study is about tracking different types of mosquitoes and the spread of the invasive Asian tiger mosquito on the remote Greek island of Kastellorizo. The research aims to understand how widespread these mosquitoes are and how prepared the local community is to deal with them. We conducted KAP (knowledge, attitude, practices) surveys, set up mosquito traps, and identified the types of mosquitoes found. The surveys revealed the need for increased public education about these health risks. The mosquito traps confirmed the presence of species such as *Aedes albopictus*, which can play a role in the spread of various diseases, along with other species like *Culex pipiens*, which is the main vector of West Nile virus. The study concludes that involving the community in mosquito monitoring is crucial in regard to helping control the mosquito population. These findings are valuable because they provide essential information for creating effective mosquito control strategies in isolated areas, ultimately helping to protect public health by reducing the risk of disease outbreaks.

**Abstract:**

The expansion of the tiger mosquito, a vector that can transmit diseases such as dengue, chikungunya, and Zika virus, poses a growing threat to global health. This study focuses on the entomological surveillance of Kastellorizo, a remote Greek island affected by its expansion. This research employs a multifaceted approach, combining KAP survey (knowledge, attitude, practices), mosquito collection using adult traps and human landing catches, and morphological and molecular identification methods. Results from questionnaires reveal community awareness and preparedness gaps, emphasizing the need for targeted education. Mosquito collections confirm the presence of the *Aedes albopictus, Aedes cretinus*, and *Culex pipiens* mosquitoes, highlighting the importance of surveillance. This study underscores the significance of community engagement in entomological efforts and proposes a citizen science initiative for sustained monitoring. Overall, this research provides essential insights for developing effective mosquito control programs in remote island settings, thereby emphasizing the importance of adopting a One Health approach to mitigate the spread of vector-borne diseases.

## 1. Introduction

As stated in the “Global Vector Control Response (GVCR) 2017–2030”, a big challenge for all societies is “the growing burden and threat of vector-borne diseases to human health” [1]. Changing climate and global connectivity are major drivers of the changing patterns of vector-borne diseases as both alter the distribution, abundance, and behavior of arthropod vectors and the pathogens they carry [2]. The mosquito vector is implicated in the transmission of several diseases that are spreading to new areas, including dengue fever, chikungunya, and the Zika virus [3,4,5,6]. Likewise, the success story of the expansion of *Ae. albopictus* in many parts of the world raises the alarm for these diseases in regions where they were previously absent [7,8].

Monitoring mosquito-borne disease risk in rural and island communities presents unique challenges [9]. The rapid spread of the two globally concerning *Aedes* species, *Ae. aegypti* and *Ae. albopictus*, is reshaping human risk in ways that remain poorly understood [10]. While considerable attention has been directed towards large urban centers, the dynamics of how these mosquito populations establish and proliferate in smaller towns and villages, which are increasingly interconnected through global trade and travel, remain understudied [11]. Entomological surveillance is an important tool for identifying new foci of disease risk [12]. By collecting data on mosquito abundance, species composition, and infection rates, valuable information can be provided in order to implement effective mosquito control programs [13]. Keeping track of the *Ae. albopictus* mosquito population through entomological surveillance is essential to monitor their spread and prevent the transmission of diseases [14,15,16]. Entomological surveillance can; however, be expensive and time intensive, especially when little is known a priori about species composition or the locations of the mosquito’s breeding habitat. Several studies have demonstrated that public questionnaires can provide useful information about perceived mosquito nuisance and resident-led control actions [17,18,19]. Similar survey methods may be effective for identifying important characteristics of mosquito exposure in new locations.

Greece is one of the European countries that has been affected by the expansion of *Ae. albopictus* in recent years [20,21]. The mosquito was first detected between 2003 and 2004, and has since spread to many parts of the country, including the Aegean islands [22]. Several studies have reported the establishment and spread of this mosquito species, which highlights the need for strengthening entomological surveillance [23,24].

Greece’s unique geography, with 114 inhabited islands [25], presents challenges for entomological surveillance due to limited access, equipment, and resources. Data on surveillance and control programs for these islands are notably sparse. By 2023, only about one-third of the islands—mostly the larger ones with better air and marine transport—have implemented such programs, leaving many smaller islands without any documented efforts. This lack of surveillance on smaller islands requires urgent attention. To address this gap, we developed a protocol for efficient data collection during brief visits to remote locations, such as Kastellorizo, Greece.

Our study focuses on evaluating the comprehensive insights provided by different types of information collected, contributing to a holistic synthesis of mosquito exposure, disease risk assessment, and potential control actions. The protocol involves (a) questionnaires to the public conducted through a door-to-door approach; (b) collection of mosquitoes using adult traps and ovitraps, providing data on both adult mosquito populations and their breeding sites; and (c) collection of mosquitoes through human landing catches (a method involving the direct interaction with humans acting as bait), offering insights into the mosquitoes’ host-seeking behavior. By employing these varied approaches, we aim to enhance our understanding of the dynamics between mosquito populations, human exposure, and the associated disease risks, ultimately informing targeted and effective control measures.

## 2. Materials and Methods

### 2.1. Study Site

Kastellorizo (or Megisti) is a small Greek island located in the southeastern Aegean Sea. It is the easternmost inhabited island of Greece and is situated roughly 2 km (1.2 miles) off the south coast of Turkey. The climate, as in the rest of Greece, is typically Mediterranean, with mild, rainy winters and warm, dry summers [26]. The island’s port operates daily, with two weekly departures specifically designated for voyages to Turkey, particularly during the summer months. The island has a total area of about 9 square kilometers (3.5 square miles) and a population of around 500 people, according to the most recent data available [25]. In 2023, the local municipal authorities recognized the necessity for a mosquito control program primarily driven by public nuisance complaints, particularly during the high tourist season. Importantly, there have been no recorded cases of vector-borne diseases on the current island. The application of the control program is focused on the management of public breeding sites, but with no existing baseline data on local species composition and mosquito abundance.

**Figure 1:**
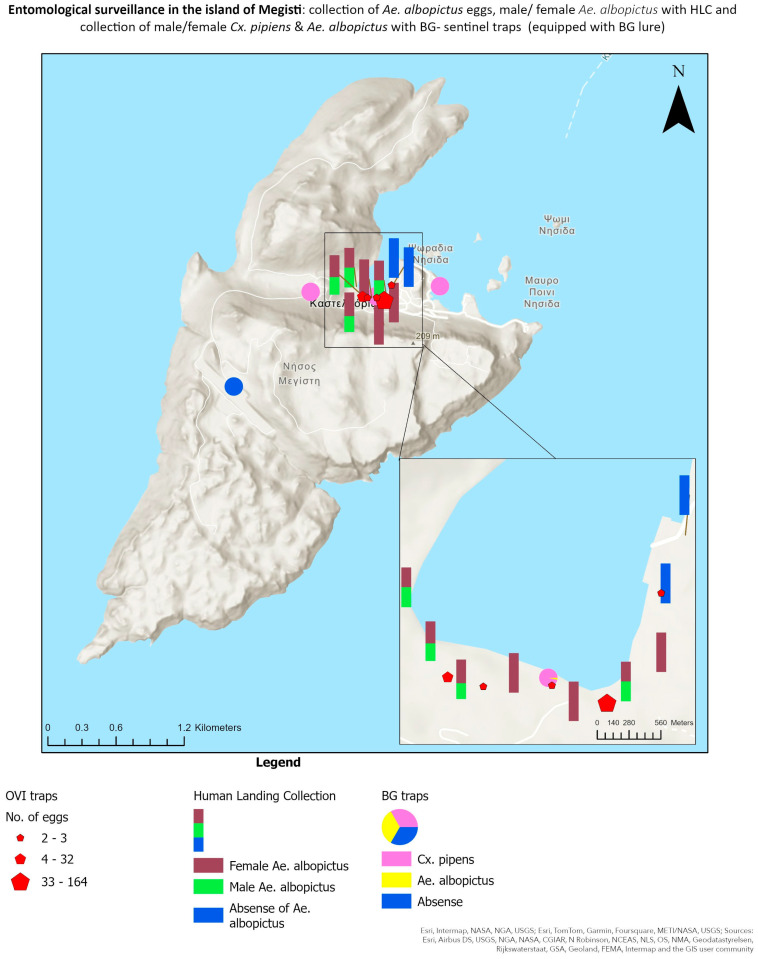
The sites of the three collection methods and the mosquito species that were collected. (Definitions “Καστελλόριζο: Kastellorizo, Νήσος Μεγίστη: Megisti Island, Ψωράδια Νησιά: Psoradia Nisia and Μαύρο Ποινι Νησιά: Mavro Poini Islet”).

**Figure 2:**
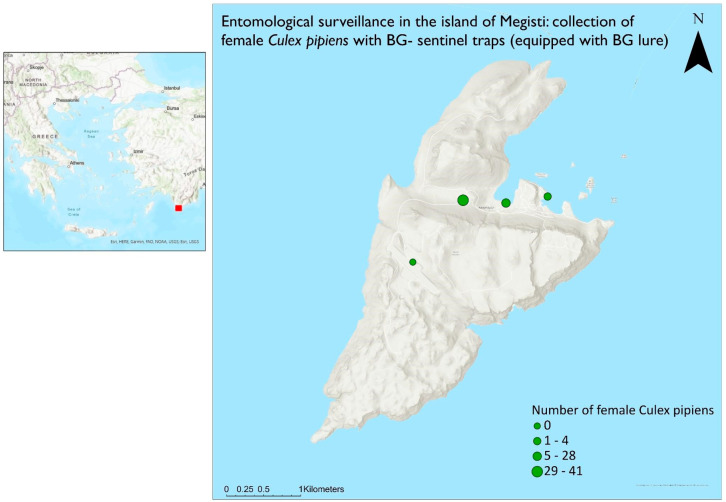
Results of the *Cx. pipiens* females for the four different locations in the island of Kastellorizo (Definitions “Καστελλόριζο: Kastellorizo and Νήσος Μεγίστη: Megisti Island”).

**Figure 3:**
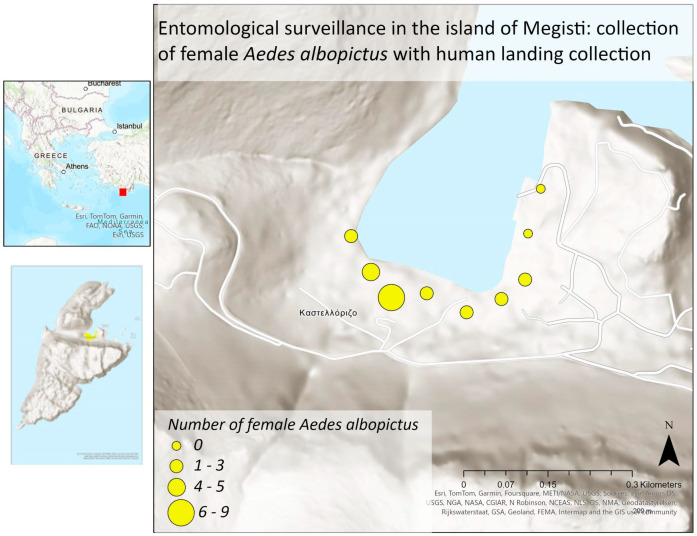
Static map of the results of the *Ae. albopictus* females for the nine different locations in the island of Kastellorizo (Definitions “Kαστελλόριζο: Kastellorizo and Νήσος Μεγίστη: Megisti Island”).

All maps (Figure 1, Figure 2 and Figure 3) were created with ArcGIS Pro (3.0.0), available from Esri (Marathon Data, GR).

### 2.2. KAP (Knowledge, Attitude, Practices) Survey

A KAP (knowledge, attitude, practices) survey was used to collect these data of local community members. The questionnaire procedure used in this study was based on previously established protocols and methods used in similar projects [19,27,28]. In more details, these protocols were carefully selected and adapted to the specific context and goals of our study (Supplementary Materials). In our study, we employed a random sampling technique to ensure representative participation. Specifically, we targeted permanent residents who frequented the coastal area during the morning hours, when various establishments like restaurants, cafes, banks, and supermarkets were bustling with activity. In selecting the singular location for questionnaire distribution, it is noteworthy that this specific site stands as the sole hub on the island, boasting a diverse array of establishments. The unique concentration of these amenities in this particular area ensures that the survey reaches a broad cross-section of the island’s population. This approach allowed us to capture a diverse cross-section of the community for our questionnaires, enhancing the robustness of our data analysis. The primary objectives of our questionnaire encompass three key goals. Firstly, it aimed to assess the presence and prevalence of mosquitoes on the island throughout the year by including questions about the active periods when mosquitoes are most commonly observed. Secondly, it sought to categorize mosquito species based on their biting behavior and preferred habitats, as different species may require distinct approaches. This information is crucial for tailoring effective control strategies. Lastly, the questionnaire aimed to evaluate the community’s readiness to adopt and implement mosquito control measures. This is vital, particularly in the event of a mosquito-related health emergency, as it provides insights into their knowledge and capacity to respond effectively.

### 2.3. Mosquito Collection

We employed three methods for collecting mosquito specimens that each target a specific life or activity stage of juvenile and adult mosquito species likely to bite humans. The specific protocols in this study targeted *Culex* and *Aedes* species, which are the main mosquito species in urban and suburban areas in Greece [29,30].

#### 2.3.1. Oviposition Traps

The selection of an entomological surveillance method is based on clear objectives, available resources, and disease prevalence. Standardization enhances data quality and enables seamless sharing and analysis [31,32]. In the case of the remote island of Kastellorizo, employing ovitraps proves promising, as it is a low-cost method and requires little expertise or training to implement. Supported by established SOPs, citizen engagement through the Mosquito Alert App, and comprehensive training resources, this approach offers an effective means to monitor and manage mosquito populations, crucial for public health [33]. In this study, from 12 to 26 July 2022, five oviposition traps (plastic cups containing 1 L water and a wooden tongue depressor) were deployed in areas with partial or full shading, with a maximum height of 50 cm from the ground. The positioning of these traps aimed to cover the widest possible area and to be in close proximity to potential mosquito breeding grounds, such as water pools and used vehicle tires left outdoors, as well as resting places such as trees and bushes. Each tongue depressor used as an oviposition substrate was marked with a unique code for identification. During collection, the tongue depressors were wrapped in damp gauze to prevent the eggs from dehydrating and were stored in transparent plastic sampling bags labeled with information about the sample, date of collection, and location [33].

#### 2.3.2. Adult Traps

To better understand the distribution and prevalence of mosquito species on the island, 4 BG-Sentinel type traps were placed in different areas. The municipality provided assistance in areas where residents had raised concerns regarding mosquito nuisance. These traps were operational for a period of 2 days (from 25 to 27 July 2022), equipped with BG-lure [34]. Each trap, powered by electricity, was strategically positioned in secure and private locations. Once the mosquitoes were caught in the trap, they were collected and stored on ice to preserve their condition for later analysis. The use of BG-lure in traps has been shown to be an effective method for monitoring mosquito populations and studying their behavior [29,30].

#### 2.3.3. Human Landing Catches

A human baiting method (HLC) was also employed to collect adult mosquitoes [35]. The task was assigned to a member of the team that approached the habitat with potential resting places for the mosquitoes, allowing them to land on their body and then using an electric aspirator to capture them. The collection was conducted during the late afternoon hours (from 6:00 p.m. to 7:00 p.m.). A total of 9 different points were selected for the sampling process, and 2 samplings (for 2 consecutive days) were carried out per point. This method was used to collect samples from different natural or artificial resting places of adult mosquitoes, including water collections, bushes, and underground areas.

The collected samples were preserved by storing them at −20 °C until their transportation to the laboratory of Insects and Parasites of Medical Importance at the Benaki Phytopathological Institute for further studies. In the lab, they were carefully examined under a stereoscope to ensure the accurate morphological identification [36,37]. In addition, the selected samples were transferred to the Laboratory of Medical Entomology, of Public Health Policy at the University of West Attica, where molecular techniques were utilized to aid in the identification of samples. This approach was particularly useful for samples that were damaged or difficult to identify based on their morphology alone.

### 2.4. Molecular Procedure

#### DNA Extraction and Polymerase Chain Reaction (PCR) Amplification

A subtotal of the collected adult mosquitoes that were morphologically identified were also examined at the molecular level to verify species identification. DNA was extracted from individual whole adults using the NucleoSpin Tissue, DNA Mini kit (MACHEREY-NAGEL, Düren, Germany), following the manufacturers’ instructions. The nuclear ribosomal spacer gene ITS2 was amplified using PCR using 5.8S and 28S primers and a part of the mitochondrial cytochrome oxidase I gene (COI) was also amplified using primers C1-J-1718 and C1-N-2191, with related PCR protocols being carried out as previously described [38,39].

Products were electrophoresed and sent for sequencing analysis (CEMIA, SA, Larissa, Greece). Similarity with sequences available in GenBank was assessed using the Basic Local Alignment Tool (BLAST+ 2.15.0, National Library of Medicine, Bethesda, MD, USA) and sequences were aligned using the CLUSTAL omega 1.2.2 software for Multiple Sequence Alignment (Clustal Omega EMBL-EBI, Hinxton, Cambridgeshire, UK). 

## 3. Results

### 3.1. Questionnaires

As previously mentioned, the primary goal of this study was to evaluate the importance of diverse information sources in constructing a comprehensive synthesis of mosquito exposure, disease risk, and potential control strategies. In total, 31 residents voluntarily and anonymously agreed to participated to the survey. In specific reference to the questionnaires, our main aim was to assess the magnitude of the human exposure to mosquitoes on the island. This objective was addressed through a targeted analysis of questions 1, 3, 9, and 14 within the questionnaire framework. Respondents ranked the numbers of mosquitoes on the island at a mean 3.32 (sd = 0.79) and their annoyance levels at 3.47 (sd = 0.94), using a ranking system that ranged from 0 for none to a 4 for excessive. Out of the total participants, 52% reported experiencing a significant exposure to mosquitoes, while 29% indicated a considerable presence and 19% reported a high number of mosquitoes in their area. The overall level of annoyance caused by mosquitoes was notably high, with 67% of participants expressing extreme annoyance and 20% reporting a great degree of irritation. Nearly all participants (90%) reported taking measures to protect themselves and their families from mosquitoes. However, a majority (70%) stated that these protective measures were insufficient and not very effective.

Our second survey objective was to identify probable species identities from respondent answers to questions about seasonal (Question 12) and diurnal (Question 2) biting habits. Analysis of the questionnaire yielded the following insights: A majority of respondents (81%) noted mosquito activity during both day and night, and the remaining respondents (19%) reported biting only day. In terms of seasonal patterns, respondents noted that mosquitoes were present nearly year-round. The average timing for when the nuisance season begins was March (numerical mean 3.12, sd = 2.08 months) and the average end reported was October-November (numerical mean = 10.5, sd = 2.25). It is noteworthy that 46% of participants indicated the onset of mosquito is-sues necessitating personal protective equipment as early as January. Furthermore, 42% of participants identified December as the month when mosquito problems typically subside.

The third goal centered on assessing the community’s preparedness. We evaluate preparedness through the respondents’ understanding or knowledge of mosquito ecology and willingness to adopt and pay for preventative behaviors. We calculated a knowledge score for each respondent that reflected correct answers to general mosquito ecology questions (Questions 4, 5, and 8) and to targeted questions about the invasive tiger mosquito (Questions 6 and 7). A perfect knowledge score of 5 signifies correct answers to all five questions. 

Respondent knowledge scores ranged from 1 to 4, with no individual scoring a perfect 5. The mean knowledge score across all surveys was 2.52 (sd = 1.03). A majority of respondents were unable to provide correct answers to questions about where mosquitoes breed (68% selected at least one wrong answer) or which sex bites (57% incorrectly chose male or both). Most (55%) were also unable to identify the invasive tiger mosquito, although nearly all (97%) knew that the tiger mosquito is a new invasive species. Additionally, the data indicated that 54% of participants accurately recognized the actual threatening insects by selecting the images of mosquitoes.

The willingness to adopt and pay for preventative behaviors was assessed through a series of questions about current practices (Questions 9-15). Regarding protective measures, a majority of participants (61%) stated that they frequently utilized natural methods, while 35% reported using chemical measures often. Only 30% of the respondents felt that their current protective measures were effective, and yet the average monthly cost reported for household mosquito protection was EUR 34 (sd = 26). The responses ranged from EUR 10–100. A majority of respondents that answered this question (74% of 23 that provided a cost estimate) reported allocating EUR 20 or more per month for personal protection, which notably exceeds the minimum wage set at EUR 780. Opinions regarding the effectiveness of mosquito population control methods were di-vided. Specifically, 20 (64%) participants reported that both natural and chemical methods were effective, while 8 (26%) and 3 (10%) participants favored chemical and natural methods, respectively. 

In terms of demographics, most participants (94%) reported living on Kastellorizo permanently, and there were more female participants (65%). The demographic information for the questionnaire responses is shown in Table 1.

### 3.2. Mosquito Collections

The data obtained from the ovitraps collections revealed that there is a well-established population of the *Ae. albopictus* present. In total, 203 eggs were collected. Detailed results of the egg collection from the traps, as well as their exact locations, are provided in Table 2. Figure 1 illustrates the distinct placement of the collection methods utilized, including ovitraps, BG traps, and HLC. Additionally, it provides an overview of the mosquito species that were collected.

The morphological identification [37] of the mosquitoes collected from the adult traps showed the presence of two mosquito species—the Asian tiger mosquito (*Ae. albopictus*) and the common mosquito *Cx. pipiens* (Table 3). In total, 79 mosquitoes (73 ♀ and 6 ♂) were *Cx. pipiens*, while only 1 ♀ belonged to *Ae. albopictus* species. Additionally, 3 mosquitoes (2 ♀ and 1 ♂) were collected that belonged to *Aedes* spp., but lacked the morphological characteristics to be identified at the species level. The common mosquito was found to be the dominant species in adult trap collections. A static map was then created to show a comparative display of the results (Figure 2).

To collect mosquitoes using the method of human landing catches, nine specific locations were selected, as indicated in Table 4. The results reveal the high activity of the Asian tiger mosquito. In total, 23 female mosquitoes and 12 male mosquitoes were collected belonging to *Ae. albopictus* species, whereas 3 (1 ♀ and 2 ♂) mosquitoes were *Cx pipiens*. Figure 3 displays a static map showing a comparative view of the results from the human landing collection of female *Ae. albopictus* mosquitoes.

### 3.3. Molecular Procedures

In order to ensure morphological identification, samples were randomly selected for molecular identification, alongside the three mosquito samples that exhibited a loss of identifiable characteristics, preventing their morphological identification to the species level. Around 25% of the morphological identified mosquitoes were tested with molecular procedures, using two different amplification protocols. These approaches were undertaken to enhance the reliability and robustness of the identification process. Amplification using PCR of ITS and COI fragments produced the expected fragments of *Ae. albopictus and Cx. pipiens*. A very interesting result was the *Ae. cretinus* samples that were identified after further analysis of the sequencing chromatograms, thus verifying the presence of both *Aedes* species in the island.

## 4. Discussion

Of the 60 species of mosquitoes that have been recorded in Greece [37,40,41], three were identified in Kastellorizo in this study. Traps were placed in several sites, representing different types of environments, and although the study was of short scale, the findings are noteworthy, since the collected species are of public health importance and worldwide distribution (*Ae. albopictus* and *Cx pipiens*). Special considerations are necessary for *Cx. pipiens* and *Ae. albopictus* due to their roles as vectors for different pathogens. *Cx. pipiens* is a primary vector of West Nile Virus, thriving in urban and semi-urban environments, often breeding in stagnant water [42]. Effective surveillance of this species is critical for monitoring potential outbreaks of arboviruses like West Nile and Usutu viruses [43,44,45]. On the other hand, *Ae. albopictus* is a significant vector of dengue, chikungunya, and Zika viruses, and is known for its adaptability to a variety of environments, including rural and urban areas [46]. This species requires surveillance, especially during the day when it is most active, and in small water sources like containers, as it poses a growing threat to public health in Europe. Both species require tailored monitoring approaches due to their distinct vectorial capacities and the diverse range of pathogens they can transmit.

Furthermore, *Ae. cretinus* was also recorded which has become scarce since the invasion of *Ae. albopictus* in Greece [47,48]. Both species, *Ae. albopictus* and *Ae. cretinus*, have been recorded together since 2003 and are known to be closely related species with common morphological features and ecological similarities [20,20,22,28,39]. It is not surprising that *Ae. albopictus* is also present in this remote island given its known ability to adapt to most types of environments and its spread and establishment in Europe so far [49]. As it is involved in pathogen transmission, its surveillance and control is of major importance for avoiding economic impacts on public health [33,50].

The recent introduction of *Ae. aegypti* in Cyprus highlights the need for comprehensive entomological surveillance not only on the island, but also in the surrounding remote areas [51]. Mosquitoes are known to travel great distances, and, as such, the introduction of *Ae. aegypti* to Cyprus may have occurred due to the movement of people or goods from neighboring countries [52,53]. Monitoring remote areas, especially those that share borders with countries where *Ae. aegypti* is endemic, is crucial to detect the presence of the mosquito early on and prevent its establishment in new areas [46,54].

Surveys in continental areas typically benefit from greater logistical support, access to a wider variety of habitats, and larger sample sizes, allowing for more robust data collection. However, small island surveys like ours can offer critical insights into isolated ecosystems and the introduction of invasive species like *Ae. albopictus*. The detection of the native mosquito *Ae. cretinus,* which has become less frequently collected since the introduction of *Ae. albopictus* in Greece, re-enforces the idea that remote areas may differ significantly from mainland regions due to geographic isolation, limited dispersal, and localized environmental factors.

While broader studies often include diverse mosquito species across varied habitats, our focus was more constrained to species like *Cx. pipiens* and *Ae. albopictus*, which are present even in isolated areas. In terms of similarities, both small- and large-scale surveys face challenges related to seasonal variations, species-specific behavior, and pathogen surveillance, but small island studies can be particularly valuable for the early detection of invasive species or emerging pathogens before they spread to larger areas [42,55].

Entomological surveillance is especially critical in remote islands where the risk of vector-borne diseases is high due to their isolation and limited access to healthcare resources [56]. In such settings, vector control strategies may be the only effective means of preventing disease transmission, including monitoring resistance status [57,58,59]. For example, in the Pacific Islands, where mosquito-borne diseases such as dengue and Zika are endemic, entomological surveillance has been a key component of successful disease control programs [60].

The results obtained from the questionnaires present a nuanced picture of the community’s preparedness in addressing the prevalent mosquito issue on Kastellorizo Island. While a substantial proportion of participants reported heightened awareness, with 90.3% actively taking measures to protect against mosquitoes, a concerning majority (70%) expressed dissatisfaction with the perceived effectiveness of these measures. These findings align with the recognition of the need for effective mosquito control strategies, emphasizing the significance of refining public education campaigns and enhancing the efficacy of protective measures [61]. The demonstrated gaps in knowledge regarding mosquito breeding sites and biting habits among participants highlight the need for targeted educational initiatives to improve community awareness, echoing the recommendations of Bartlett-Healy et al. (2012) [62]. The financial commitment to personal protection, exceeding the minimum wage for a majority (73.9%) of participants, emphasizes the economic burden imposed by the mosquito issue, reinforcing the importance of sustainable mosquito control strategies [63].

These findings underscore the critical importance of engaging the community in entomological surveillance and control efforts. In line with recent research [27,28], fostering a more informed and unified community approach to mosquito control is paramount for fortifying the island’s preparedness and resilience against the persistent challenge of mosquitoes. Furthermore, this study’s insights into the pivotal role of public awareness emphasize the integral nature of community support and participation in ensuring the success and sustainability of entomological surveillance and control programs [64]. Effective communication strategies, as highlighted by Wong et al. (2014) [65], become instrumental in enhancing public knowledge and participation in vector control efforts, further supporting the need for comprehensive and community-centric approaches to address mosquito-related challenges on Kastellorizo Island.

We are aware that we could only deploy a limited number of ovitraps and adult traps, which restricted our data collection. Due to our short visit to the remote island of Kastellorizo, the resulting dataset is smaller and may not fully represent the island’s mosquito population. Similarly, the limited time affected our ability to gather extensive responses for the questionnaires. Although our goal was to quickly understand the mosquito situation, the small sampling size may have impacted the comprehensiveness of our findings. Future studies could strengthen surveillance by incorporating larval surveys using dippers and by exploring crab holes for *Deinocerites* mosquitoes, as well as by sampling rock holes and habitats suitable for *Anopheles* species. It is important to highlight that there were no existing baseline data on local species composition and mosquito abundance, primarily due to the remote nature of the current island. Our small-scale study underscores the significance of implementing surveillance and data collection measures as integral components of the control initiative. The results obtained from this small-scale study serve both as preliminary insights and as a foundation for more extensive vector studies that will cover a more extended period and include additional sampling sites. The data obtained from this study can highlight the significance of conducting entomological studies in remote areas, and the knowledge gained is valuable in contributing to the implementation of mosquito management programs and public health actions. As future actions, we are in the process of designing a tailored approach for a citizen science initiative, utilizing the Mosquito Alert phone application [66,67,68]. This approach aims to actively involve the community in mosquito monitoring, fostering a collaborative effort in the management and control of mosquito-related issues on the island.

## Figures and Tables

**Table 1:** Questionnaire responses (*n* = 31) on demographics.

Demographic Information	Number of Responses (%)
Sex	
Woman	20 (65)
Man	11 (35)
Type of settlement	
Permanent residence	29 (94)
Holiday residence	2 (6)

**Table 2:** Information regarding the ovitrap collections in the five different locations in the island of Kastellorizo.

Ovitraps	Lat	Long	Installation Day	Collection Day	No. of Eggs
K1	36.149806°	29.592298°	12 July 2022	26 July 2022	2
K2	36.149044°	29.589423°	12 July 2022	26 July 2022	32
K3	36.148920°	29.589885°	12 July 2022	26 July 2022	3
K4	36.148889°	29.590789°	12 July 2022	26 July 2022	2
K5	36.148671°	29.591501°	12 July 2022	26 July 2022	164

**Table 3:** Information regarding the adult collections in the four different locations on the island of Kastellorizo.

Adult Traps	Location	Lat	Long	Installation Day	Collection Day	*Cx. pipiens*		*Ae. albopictus*		*Ae. cretinus*	
						♀	♂	♀	♂	♀	♂
BG1	Airport	36.142480°	29.576363°	25 July 2022	27 July 2022	0	1	0	0	0	0
BG2	Public infrastructure	36.149609°	29.584378°	25 July 2022	27 July 2022	41	2	0	0	0	0
BG3	Mandraki	36.149468°	29.597007°	25 July 2022	27 July 2022	4	0	0	0	2	1
BG4	Square (Lazarakis)	36.148968°	29.590747°	25 July 2022	27 July 2022	28	3	1	0	0	0

**Table 4:** Locations where adult mosquitoes were collected using the human landing collection method and the recorded species identification results.

HLC	Lat	Long	Collection Day	*Cx. pipiens*		*Ae. albopictus*	
				♀	♂	♀	♂
K_HLC1	36.149822°	29.588870°	25 July 2022	0	0	1	1
K_HLC2	36.149230°	29.589221°	25 July 2022	1	2	2	3
K_HLC3	36.148804°	29.589596°	25 July 2022	0	0	0	0
K_HLC4	36.148840°	29.590294°	25 July 2022	0	0	0	0
K_HLC5	36.148499°	29.591061°	25 July 2022	0	0	0	0
K_HLC6	36.148679°	29.591763°	25 July 2022	0	0	0	0
K_HLC7	36.148969°	29.592251°	25 July 2022	0	0	0	0
K_HLC8	36.149701°	29.592360°	25 July 2022	0	0	0	0
K_HLC9	36.150406°	29.592660°	25 July 2022	0	0	0	0
K_HLC1	36.149822°	29.588870°	26 July 2022	0	0	0	0
K_HLC2	36.149230°	29.589221°	26 July 2022	0	0	3	1
K_HLC3	36.148804°	29.589596°	26 July 2022	0	0	9	6
K_HLC4	36.148840°	29.590294°	26 July 2022	0	0	3	0
K_HLC5	36.148499°	29.591061°	26 July 2022	0	0	2	0
K_HLC6	36.148679°	29.591763°	26 July 2022	0	0	1	1
K_HLC7	36.148969°	29.592251°	26 July 2022	0	0	2	0
K_HLC8	36.149701°	29.592360°	26 July 2022	0	0	0	0
K_HLC9	36.150406°	29.592660°	26 July 2022	0	0	0	0

## Data Availability

All data are available in the manuscript.

## Supplementary Materials

The following supporting information can be downloaded at: https://www.mdpi.com/article/10.3390/insects15090724/s1.
