# Supplementary material for: Mitigating the Threat of Invasive Mosquito Species Expansion: A Comprehensive Entomological Surveillance Study on Kastellorizo, a Remote Greek Island
Source: Insects. 2024 Sep 20;15(9):724. doi: 10.3390/insects15090724 (PMC11432031; doi:10.3390/insects15090724)
Supplement: Supplementary file 1 [file insects-15-00724-s001.zip › insects-3192243-supplementary.pdf]

Article

Mitigating the Threat of Invasive Mosquito Species Expansion: A Comprehensive Entomological Surveillance Study on Kastellorizo, a Remote Greek Island

Marina Bisia [1,†], Georgios Balatsos [1,†], Stavroula Beleri [2], Nikolaos Tegos [2], Evangelia Zavitsanou [1], Shannon L. LaDeau [3], Vasilis Sotiroudas [4], Eleni Patsoula [2] and Antonios Michaelakis [1,*]

[1] Laboratory of Insects and Parasites of Medical Importance, Scientific Directorate of Entomology and Agricultural Zoology, Benaki Phytopathological Institute, 145 61 Kifissia, Greece; m.bisia@bpi.gr (M.B.); g.balatsos@bpi.gr (G.B.); e.zavitsanou@bpi.gr (E.Z.)
[2] Laboratory for the Surveillance of Infectious Diseases, Department of Public Health Policy, School of Public Health, University of West Attica, 115 21 Athens, Greece; smpeleri@uniwa.gr (S.B.); ntegos@uniwa.gr (N.T.); epatsoula@uniwa.gr (E.P.)
[3] Cary Institute of Ecosystem Studies, Millbrook, NY 12545, USA; ladeaus@caryinstitute.org
[4] AgroSpeCom, 7th klm National Road Thessaloniki-Katerini, Kalochori, 570 09 Thessaloniki, Greece; v.sotiroudas@agrospecom.gr
* Correspondence: a.michaelakis@bpi.gr
† These authors contributed equally to this work.

Supplementary material – KAP Questionnaire

KAP Questionnaire – Kastellorizo (English version)

1. **Are there any mosquitoes in your area?**

0	1	2	3	4
No	A few	Many	Quite a lot	Excessive

2. In case of a positive answer, then they are mostly seen:

During daytime	
During night	
Both	

3. **Level of your annoyance:**

0	1	2	3	4
None	A little	Some	Great	Extreme

4. **Where do the mosquitoes lay eggs?**

Standing water		Mud		Grass-plants	

Soil		Flowerpot		Creeks	
Drainage		Old (used) tires		External flowerpots	

5. **Do you know which mosquitoes bite?**

Males		Females		Both	

6. **The Asian mosquito tiger is considered:**

Local species		New, non-local species	

7. **Which of the following is the mosquito 'tiger'?**

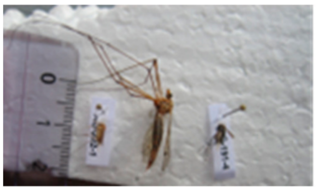

1. □ 2. □ 3. □

8. **Which of the above is more threatening?**

□	□	□	□
None	the first	the second	the third

9. **Do you or any member of your family take measures of protection against mosquitoes?**

□ □
Yes. No

10. **When you want to be protected from mosquitoes on your property, how often do you choose natural measures of protection (e.g. mesh, mosquito net, fan) ?**

0	1	2	3	4
Never	Rarely	Sometimes	Often	Very often

11. **Respectively, when you want to be protected from mosquitoes on your property, how often do you choose measures of protection that are based on synthetic chemical insecticides?**

0	1	2	3	4
Never	Rarely	Sometimes	Often	Very often

12. **During which months do you typically encounter mosquito issues that require the use of personal protective equipment, either natural or chemical?**

Start month of mosquito problem.:..........

End month of mosquito problem:............

13. **During the above-mentioned time frame, what is the average monthly household expenditure on measures to combat the mosquito problem? ....(open question)**

14. **When you take measures of protection against mosquitoes, do you feel that they are sufficient and effective?**

☐ ☐
Yes. No

15. **Which methods of mosquitoes' population control, is more effective?**

Natural measures of protection		Synthetic chemical insecticides		Both	

16. **You live:**

☐ ☐
Permantly For holidays

17. **Sex**

☐ ☐ ☐
Male Female Without answer
